# Arthritis Awareness Month — May 2013

**Published:** 2013-05-03

May is Arthritis Awareness Month. Arthritis affects an estimated 50 million U.S. adults (1) and continues to be the most common cause of disability in the United States (2). This year’s theme, “Faces of Arthritis,” ( http://www.arthritis.org/facesofarthritis ) is designed to challenge arthritis stereotypes and educate the public about the impacts of arthritis, along with promoting clinical and public health interventions to control it.

Common arthritis stereotypes suggest that arthritis only affects older adults and that it is inevitable and untreatable. However, arthritis can affect persons at any age, including children, and most persons with arthritis are aged <65 years (3). Further, arthritis comprises a set of diseases that are not a normal part of aging. Even after arthritis is diagnosed, there are many measures that can minimize disease progression and joint pain as well as help patients maintain function. For example, persons with arthritis can supplement clinical management with physical activity, which reduces arthritis pain and helps manage coincident problems, such as diabetes, heart disease, and obesity (4). In addition, self-management education helps persons with arthritis gain control of their condition by learning techniques to manage their symptoms and reduce pain and activity limitations (5).

Information about ways to help manage arthritis is available at http://www.cdc.gov/arthritis. Additional information is available from the Arthritis Foundation ( http://www.arthritis.org ) and the National Institute of Arthritis and Musculoskeletal and Skin Diseases ( http://www.nih.gov/niams ).
